# Rapid Visual LAMP-CRISPR/Cas12a Detection System for *Cytospora* Pathogens Associated with Pear Canker Integrating Isothermal Amplification with Programmable Secondary Recognition

**DOI:** 10.3390/jof12100743

**Published:** 2026-10-03

**Authors:** Pan Xie, Canpeng Fu, Lingkai Xu, Jiaqi Ding, Beibei Feng, Shuaishuai Sha

**Affiliations:** 1 School of Advanced Agricultural Sciences, Kashi University, Kashi 844000, China; panxie126@sina.com (P.X.); fucp000@outlook.com (C.F.);; 2 Research Center for Crop Biotechnology Breeding and Smart Cultivation in Southern Xinjiang, Kashi 844000, China

**Keywords:** LAMP, CRISPR/Cas12a, *Cytospora pyri*, *Cytospora mali*, pear canker, fluorescence detection, field testing

## Abstract

Pear canker, caused by *Cytospora* pathogens, is a devastating branch and trunk disease threatening the sustainable development of the fragrant pear industry in Korla, Xinjiang, China. Owing to the pathogen’s latent infection capability and the inconspicuous nature of early symptoms, symptom-based diagnosis is inadequate for early warning and precision management. Here, we developed a hybrid LAMP-CRISPR/Cas12a fluorescence detection system targeting *Cytospora pyri* and its closely related congener *C. mali*, the dominant *Cytospora* pathogens associated with pear canker. LAMP primers and Cas12a crRNA recognition sites were designed from conserved sequences of the β-tubulin (*TUB2*) gene, enabling rapid isothermal amplification and sequence-specific secondary confirmation. The optimized LAMP module amplifies the target within 30 min at 65 °C, and the CRISPR/Cas12a module produces stable fluorescence after approximately 15 min of trans-cleavage at 37 °C. The system yields positive signals for both *C. pyri* and the closely related *C. mali*, with no cross-reactivity against other *Cytospora* species or common fruit tree pathogenic fungi, supporting rapid screening of pome fruit canker pathogens. The limit of detection is 1 copy/μL of target template. Validation using *Cytospora* strains from diverse geographic and host origins demonstrated complete concordance between LAMP gel electrophoresis, CRISPR/Cas12a real-time fluorescence detection, and UV-assisted visual evaluation. Preliminary results from field-collected Korla fragrant pear branch and trunk samples were consistent with conventional PCR, supporting applicability to orchard samples. By integrating rapid isothermal amplification, secondary sequence verification, and simple visual evaluation, this system provides a robust platform for early screening, pathogen surveillance, and precision management of *Cytospora* canker in Korla fragrant pear orchards.

## 1. Introduction

Species of the genus Cytospora are major fungal pathogens of woody plants, causing branch and trunk cankers on economically important Rosaceae fruit trees, including apple (*Malus domestica*), pear (*Pyrus* spp.), peach (*Prunus persica*), and apricot (*Prunus armeniaca*). These diseases result in substantial economic losses to orchard industries worldwide [1,2,3]. Infection occurs through wounds or natural openings and progresses to bark discoloration, necrosis, branch dieback and girdling cankers that may kill the tree, the disease is characterized by latent infection and episodic outbreaks [4]. Korla fragrant pear (*Pyrus sinkiangensis* Yu) is a flagship crop and pillar of the specialty fruit industry in southern Xinjiang, China [5], and chronically affected by pear canker, which undermines tree vigor and forces orchard renewal. The pathogen assemblage associated with pear canker in this region is dominated by *Cytospora* species (formerly *Valsa* spp.), whose composition varies among hosts and production regions [6]. Of these, *C. pyri* has been demonstrated to be pathogenic to fragrant pear, penetrating shoots through wounds, colonizing bark tissues and inducing typical cankers [3,7], and it is the primary target of this study together with its closely related congener *C. mali*. Because infected trees can remain asymptomatic while the pathogen persists in bark and xylem, diagnosis based on visible lesions is inadequate for detecting incipient infections; rapid and sensitive detection of the dominant *Cytospora* pathogens before symptom expression is therefore essential for early warning and precision management [8].

Current control of pear canker in fragrant pear orchards still relies largely on symptom-based management, namely lesion excision, pruning of infected branches and application of chemical fungicides [9]. These are labor-intensive methods that eradicate latent pathogens already residing in woody tissues, so shifting from post-disease treatment to early pathogen identification and risk monitoring would enable timely preventive interventions [10,11]. Existing diagnostic methods have their limitations. Culturing coupled with morphological identification can isolate and tentatively confirm the pathogen but is slow and sensitive to culture conditions, morphological variability and subjective interpretation [12,13]. Immunoassays such as ELISA and lateral-flow devices are rapid, but development of pathogen-specific antibodies is protracted and analytical sensitivity is vulnerable to sample-matrix effects [14,15]. PCR and qPCR are sensitive and accurate, but they depend on thermal-cycling instrumentation and demanding sample preparation, which limits their use for on-site screening in resource-limited orchards [16,17].

Isothermal amplification techniques, notably loop-mediated isothermal amplification (LAMP) and recombinase polymerase amplification (RPA), obviate the need for thermal cycling and offer rapid reaction kinetics with reduced instrumentation demands [18,19,20]. LAMP uses multiple primers that recognize distinct regions of the target sequence, providing high amplification efficiency, short reaction times and potential for minimal-instrumentation visual evaluation [21,22]. However, this multi-primer configuration increases the risk of primer–dimer formation and non-specific amplification, which can generate false-positive results and compromise diagnostic reliability [23,24]. A secondary, sequence-specific recognition step is therefore required to confirm the identity of the LAMP amplicon.

CRISPR/Cas systems are versatile, programmable nucleic-acid detection platforms [25,26,27]. Guided by CRISPR RNA (crRNA) and requiring a 5′-TTTV-3′ protospacer adjacent motif (PAM), Cas12a activates collateral trans-cleavage of a fluorophore/quencher-labeled single-stranded DNA (ssDNA) reporter upon target recognition, thereby converting sequence-specific recognition into a fluorescent and visually interpretable signal [28,29]. Coupling CRISPR/Cas12a with isothermal amplification yields rapid and highly specific detection without thermal-cycling equipment [30,31]. While RPA-CRISPR/Cas12a has been applied to plant pathogens, LAMP offers lower reagent cost, more robust reaction conditions and greater suitability for large-scale orchard screening, and combining LAMP with Cas12a retains operational convenience while mitigating the false-positive risk of standalone LAMP [32,33]. LAMP-CRISPR/Cas12a systems have already been used to detect plant viruses and other pathogens, illustrating the broad applicability of this integrated approach [34,35].

This study aimed to develop and evaluate a two-stage LAMP-CRISPR/Cas12a assay for group-level screening of C. pyri and C. mali associated with pear canker, thus providing an instrument-light, sequence-verified tool for early detection and orchard-level disease surveillance.

## 2. Materials and Methods

### 2.1. Fungal Isolation and DNA Extraction

A total of 32 fungal strains were selected for this study, including 20 strains of *C. pyri*, 5 strains of *C. mali,* and the closely related species *Cytospora parasitica*, *Cytospora chrysosperma* and *Cytospora melnikii*, as well as the non-target fungal species commonly found in the Xinjiang region: *Fusarium oxysporum*, *Fusarium verticillioides*, *Botryosphaeria dothidea* and *Alternaria alternata*. All strains used in this study were provided by thePlant Pathology Laboratory at Kashi University, China (Table 1).

To extract genomic DNA, purified isolates were grown on PDA at 25 °C for 7 days. Fresh mycelia were harvested using a sterile scalpel or inoculation needle and subjected to mechanical disruption using an automated sample grinder (JXFSPRP-48L; JingXin, Shanghai, China). Total genomic DNA was extracted using a fungal genomic DNA extraction kit (B518229; Sangon Biotech, Shanghai, China) according to the manufacturer’s protocol. The concentration and purity of extracted DNA were assessed using a UV–Vis spectrophotometer (UV1700; Lengguang Technology, Shanghai, China) and quality was evaluated based on A260/A280 and A260/A230 absorbance ratios. DNA samples meeting quality criteria were stored at −20°C.

### 2.2. Primer and crRNA Design

A *TUB2* gene fragment was selected as the candidate target region to satisfy the requirements of both LAMP and Cas12a target recognition. β-tubulin sequences of *C. pyri* and related *Cytospora* species—*C. mali*, *C. chrysosperma*, *C. tritici*, *C. melnikii*, *C. euonymina*, *C. parasitica*, and *C. donglingensis*—were retrieved from the NCBI GenBank database and combined with *TUB2* sequences from representative strains in this study obtained previously. Multiple sequence alignment was performed to evaluate sequence conservation in the target region and interspecific divergence among congeneric species. Based on alignment results, a *TUB2* fragment suitable for LAMP primer arrangement and containing a recognizable Cas12a PAM site was selected as the candidate design region.

LAMP primers were designed using PrimerExplorer V5 (https://primerexplorer.eiken.co.jp/lampv5e/index.html, accessed on 12 February 2026) [36]. Comprehensive design parameters included melting temperature (Tm), GC content, primer spacing, dimer formation potential, and secondary structure stability of outer, inner, and loop primers. Candidate primers were subjected to preliminary evaluation of non-specific amplification using Primer-BLAST (https://www.ncbi.nlm.nih.gov/tools/primer-blast, accessed on 12 February 2026). The final LAMP primer set used for subsequent experiments comprised outer primers F3 and B3, inner primers FIP and BIP, and loop primer LF. The inner primers FIP and BIP are chimeric: FIP consists of the F1c sequence (complementary to F1) linked to the F2 sequence via a TTTT spacer, and BIP consists of the B1c sequence linked to the B2 sequence. This chimeric design is essential for the loop-mediated amplification mechanism and accounts for the greater length of FIP and BIP relative to the outer primers (Table 2). All primers were synthesized by Tsingke Biotechnology Co., Ltd. (Xi’an, China).

Cas12a crRNAs were designed using the CHOPCHOP online tool (https://chopchop.cbu.uib.no) [37]. The *TUB2* fragment within the LAMP-amplified region was used as the input sequence, with the CRISPR/Cas12a design module selected and the PAM constraint set to 5′-TTTV-3′ (where V represents A, C, or G). Candidate crRNAs were screened based on PAM position, target sequence conservation, GC content, and predicted off-target potential. To enable fluorescence signal generation upon Cas12a activation, an ssDNA reporter dual-labeled with a fluorophore (FAM) and a quencher (BHQ1) at the 5′ and 3′ termini, respectively, was designed. Sequences of the crRNAs and ssDNA reporter are listed in Table 3.

### 2.3. LAMP-CRISPR/Cas12a Assay Optimization

In establishing the hybrid LAMP-CRISPR/Cas12a detection system, the LAMP and CRISPR/Cas12a fluorescence detection modules were optimized separately. Genomic DNA of *C. pyri* was used as the template for LAMP optimization, with the amplification products being used as templates for CRISPR/Cas12a optimization. All optimization experiments used nuclease-free water as a negative control and three independent replicates per condition.

The LAMP reaction was performed in a total volume of 25 μL comprising 2.5 μL of 10× Isothermal LAMP Buffer, 3.5 μL of dNTPs (10 mM), 2.5 μL of 10× LAMP Primer Mix, 1.0 μL of Bst 2.0 DNA polymerase (8 U/μL), 2.0 μL of betaine (5 M), 1.5 μL of MgSO_4_ (100 mM), 3.0 μL of DNA template, and 9.0 μL of nuclease-free water. The 10× LAMP Primer Mix contained outer primers F3 and B3, inner primers FIP and BIP, and loop primer LF. When added to the 25 μL reaction mixture, the final concentrations were 0.2 μM F3 and B3, 1.6 μM FIP and BIP, and 0.4 μM LF, yielding an outer:inner:loop primer ratio of 1:8:2. The negative control substituted nuclease-free water for the DNA template.

LAMP was conducted in a thermostatic water bath (LICHEN, Shanghai, China). Following amplification, the reaction was terminated by heat inactivation at 80 °C for 10 min. Amplification products were analyzed using 1.2% agarose gel electrophoresis at 180 V for 15 min, with the resulting bands being visualized in a gel documentation system (Tanon-1600; Tanon, Shanghai, China).

LAMP reaction conditions were optimized using various gradient series: Reaction temperatures 61, 62, 63, 64, 65, 66, 67, 68, and 69 °C; reaction times 15, 20, 30, and 40 min; outer:inner:loop primer concentration ratios 1:6:2, 1:6:3, 1:6:4, 1:8:2, 1:8:3, 1:8:4, 2:8:3, and 2:6:3; and final dNTP concentrations 0.8, 1.0, 1.2, 1.4, 1.6, 1.8, and 2.0 mM. Amplification products were assessed under each condition using agarose gel electrophoresis, with the optimization criteria being band clarity, amplification intensity, and absence of amplification in negative controls.

LAMP products were subsequently used as templates for CRISPR/Cas12a fluorescence detection. The CRISPR/Cas12a reaction was performed in a total volume of 20 μL comprising 2.0 μL of 10× Cleavage Buffer, 1.0 μL of LbCas12a (10 μM; 0.5 μM final concentration), 1.0 μL of crRNA (10 μM; 0.5 μM final concentration), 4.0 μL of ssDNA reporter (10 μM; 2.0 μM final concentration), 2.0 μL of LAMP amplification product (added directly without dilution), and 10.0 μL of nuclease-free water. The initial molar ratio of LbCas12a to crRNA was 1:1. The negative control substituted nuclease-free water for the LAMP amplification product.

The CRISPR/Cas12a reaction was conducted in a real-time quantitative PCR system (Q2000B; Long Gene, Hangzhou, China), with fluorescence signals acquired using the FAM channel. Following the reaction, the mixture was transferred to a transparent 200 μL centrifuge tube and examined under a UV lamp (365 nm) to visually evaluate the fluorescence signal.

CRISPR/Cas12a reaction conditions were optimized using various gradient series: Reaction temperatures 35, 37, 39, 41, 43, and 45 °C; reaction times 5, 10, 15, 20, 30, 40, and 50 min; LbCas12a final concentrations 0.25, 0.50, 0.75, and 1.00 μM; LbCas12a:crRNA molar ratios 1:0.5, 1:1, 1:2, 1:3, and 1:4; and ssDNA reporter final concentrations 250, 500, 750, 1000, 1250, 1500, and 2000 nM. Conditions were evaluated based on real-time fluorescence curves, endpoint fluorescence intensity, and fluorescence signals visible under UV light.

### 2.4. Specificity of the LAMP-CRISPR/Cas12a System

To evaluate the analytical specificity of the hybrid system, a panel of fungal isolates was assembled, comprising the target pathogen *C. pyri*, closely related congeneric *Cytospora* species, and non-target fungi commonly associated with branch and trunk diseases of fragrant pear and other fruit trees in Xinjiang. The tested isolates were *C. pyri*, *C. mali*, *C. parasitica*, *C. chrysosperma*, *C. melnikii*, *F. oxysporum*, *F. verticillioides*, *B. dothidea*, and *A. alternata*. *C. pyri* DNA served as the positive control and nuclease-free water was substituted for DNA in the negative control.

Specificity testing was performed using the optimized LAMP and CRISPR/Cas12a reaction conditions. LAMP products were analyzed using 1.2% agarose gel electrophoresis. An aliquot of each LAMP product was added to the CRISPR/Cas12a reaction mixture for real-time fluorescence detection, followed by visual examination under UV light. Each analysis was carried out on three independent replicates.

Positive results were defined as the simultaneous fulfillment of three criteria: (i) the presence of characteristic ladder-like bands on the agarose gel, indicative of successful LAMP; (ii) real-time fluorescence signals from the CRISPR/Cas12a reaction that markedly exceeded the negative control; and (iii) visible fluorescence under UV light. A negative result was assigned when neither typical LAMP ladder-like bands nor a CRISPR/Cas12a fluorescence signal above the negative control threshold were observed.

### 2.5. Sensitivity of the LAMP-CRISPR/Cas12a System

To evaluate the analytical sensitivity of the hybrid system, genomic DNA extracted from pure cultures of *C. pyri* was used as the standard template. DNA was extracted using a fungal genomic DNA extraction kit (B518229; Sangon Biotech, Shanghai, China). Following quantification of concentration and purity, target-gene copy number was derived from the measured DNA mass concentration under the assumption that *TUB2* is a single-copy gene in the haploid genome. This assumption is supported by the finding that the β2-tubulin paralog is lost in most ascomycetes, leaving a single β-tubulin locus [38], and is consistent with the single β-tubulin locus annotated in the published *C. pyri* and *C. mali* genomes [39]. Using the haploid genome size 35.7 Mb reported for *C. pyri* (*V. pyri*) (44.7 Mb for *C. mali*/*V. mali)* by Yin et al. [39], the copy-number concentration was calculated as: copies/μL = (DNA mass in g × 6.022 × 10^23^)/(genome length in bp × 660 g·mol^−1^·bp^−1^), where 660 g·mol^−1^·bp^−1^ is the mean molecular weight of one double-stranded base pair.

Each dilution was used as a template in the optimized LAMP reaction. Following amplification, a portion of the product was analyzed using 1.2% agarose gel electrophoresis and the remainder was subjected to CRISPR/Cas12a real-time fluorescence detection using UV-assisted visual examination. Sterile water was substituted for DNA in the negative control.

Three independent replicates were performed for each template concentration. Positive results were defined as the concurrent fulfillment of three criteria: (i) characteristic ladder-like bands indicative of successful LAMP; (ii) CRISPR/Cas12a real-time fluorescence signals markedly exceeding the negative control; and (iii) visible fluorescence under UV light. The limit of detection (LOD) was defined as the lowest template concentration within the tested range that consistently yielded positive results across all replicates.

### 2.6. Applicability of LAMP-CRISPR/Cas12a System Across Diverse Cytospora Strains

To evaluate the applicability of the LAMP-CRISPR/Cas12a system across diverse *Cytospora* strains, eight *Cytospora* strains previously identified as pathogens associated with pear canker were selected. The strains, designated Cpf-043, Cpf-051, Cpf-056, Cpf-067, Cpf-029, Cpf-031, Lkx-008, and Lkx-017, varied in geographic origin, host, and collection site. The optimized LAMP and CRISPR/Cas12a reaction conditions were applied. LAMP products were analyzed using 1.2% agarose gel electrophoresis. An aliquot of each product was then subjected to CRISPR/Cas12a real-time fluorescence detection with visual examination under UV light. Each strain was tested in three independent replicates.

Detection capability of the system across the diverse *Cytospora* isolates was based upon agreement between LAMP gel electrophoresis, CRISPR/Cas12a real-time fluorescence, and UV-assisted visual assessment results, a concordance criterion commonly used to validate nucleic-acid-based diagnostic assays [35,40].

### 2.7. Field Validation of the LAMP-CRISPR/Cas12a System

To evaluate the practical applicability of the LAMP-CRISPR/Cas12a system to real-world orchard samples, one branch tissue sample each was collected from each of 20 fragrant pear trees distributed across commercial orchards in Kashi, Xinjiang Uygur, China, with the symptom status of each sample being recorded. Genomic DNA was extracted from these field samples using a modified polyethylene glycol (PEG)–sodium acetate precipitation method that was adapted from previously reported protocols to enhance inhibitor removal from bark tissues [40,41,42,43]. This method incorporated three buffer solutions: (A) sorbitol pre-wash solution (100 mM Tris-HCl, 0.35 M sorbitol, 5 mM EDTA, 0.5% β-mercaptoethanol, pH 8.0), (B) CTAB lysis buffer (100 mM Tris-HCl, 1.4 M NaCl, 20 mM EDTA, 2% cetyltrimethylammonium bromide [CTAB], 2% polyvinylpyrrolidone-40 [PVP-40], 0.5% β-mercaptoethanol, 100 μg/mL proteinase K, pH 8.0), and (C) PEG–sodium acetate precipitation solution (18% PEG 8000, 0.25 M sodium acetate, pH 5.2).

The extraction procedure was as follows. Bark tissues of appropriate size were excised in the field, surface-sterilized by sequential immersion in 70% ethanol and 3% sodium hypochlorite, then rinsed thoroughly with sterile distilled water. The sterilized tissues were flash-frozen in liquid nitrogen and ground to a fine powder using a mortar and pestle. Approximately 100 mg of powdered sample was transferred to a 2 mL microcentrifuge tube and 1 mL of ice-cold Solution A was added. The mixture was incubated on ice for 10 min with intermittent inversion (2–3 times), followed by centrifugation at 12,000× *g* for 2 min. The supernatant was discarded and 500 μL of preheated Solution B (65 °C) was added to the pellet. The mixture was vortexed thoroughly and incubated at 65 °C for 60 min with gentle inversion every 10 min. After cooling to room temperature, an equal volume of chloroform:isoamyl alcohol (24:1, *v/v*) was added and the tube was inverted repeatedly to mix. This mixture was centrifuged at 12,000× *g* for 10 min, and the upper aqueous phase was transferred to a new microcentrifuge tube. An equal volume of Solution C was added to the aqueous phase and the mixture was incubated at 4 °C for 2 h to precipitate nucleic acids. Following centrifugation at 12,000× *g* for 10 min, the supernatant was discarded and the pellet was washed twice with 500 μL of 70% ethanol (resuspending by inversion and centrifuging at 12,000× *g* for 5 min each time). The pellet was air-dried at room temperature for 10 min with the tube cap open, then dissolved in 50 μL of TE buffer supplemented with RNase A (final concentration 50 μg/mL).

The extracted DNA was used as the template in three parallel detection assays: conventional PCR, LAMP, and LAMP-CRISPR/Cas12a detection with UV-assisted visual assessment. Given the specific architecture of LAMP primers, the outer LAMP primers F3 and B3 were used as the forward and reverse primers for conventional PCR, following the design strategy of Zhou et al. [44]. The PCR program consisted of an initial denaturation step of 5 min at 94 °C, followed by 35 cycles of 30 s at 94 °C, 30 s at 55 °C and 30 s at 72 °C, and a final extension step of 10 min at 72 °C. Genomic DNA from the known positive *C. pyri* strain Cpf-043 (GenBank accession PZ629081) served as the positive control and nuclease-free water as the negative control. Agreement between the LAMP-CRISPR/Cas12a and conventional PCR results was evaluated to assess the reliability of the hybrid system for field sample analysis.

### 2.8. Statistical Analysis

Significance levels are indicated by * *p* < 0.05, ** *p* < 0.01, and *** *p* < 0.001, while ns (not significant) indicates no statistically significant difference. All comparisons were based on a single one-way ANOVA followed by Tukey’s test with a decision threshold of *p* < 0.05 (the graded asterisks indicate the level of significance and do not represent separate statistical tests).

## 3. Results

### 3.1. LAMP Primer and crRNA Design

**Figure 1:**
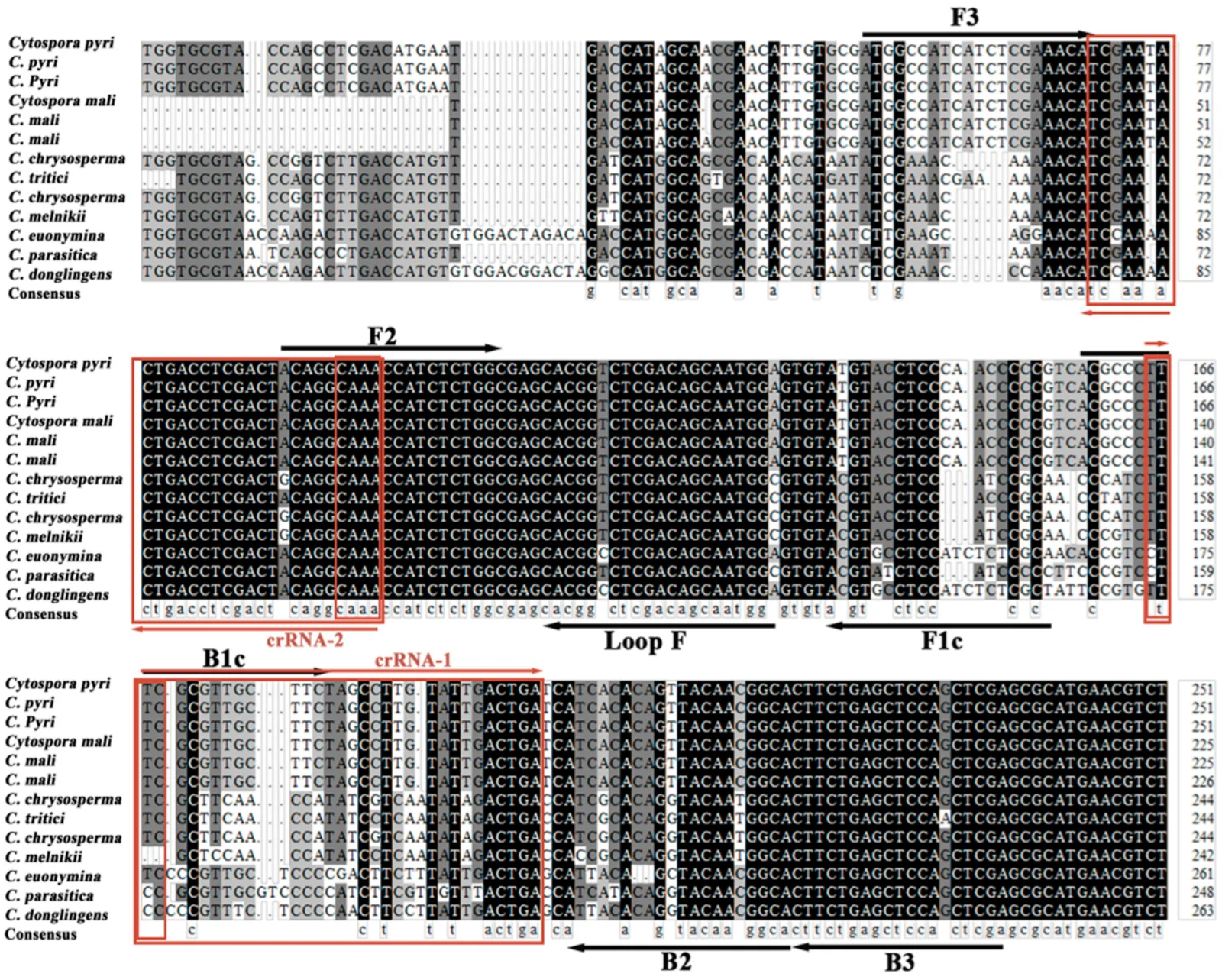
Design of LAMP primers and Cas12a crRNA target sites within the β-tubulin target region.

Sequence characteristics of the target region and the quality of primer design directly influence amplification efficiency and detection specificity of the LAMP-CRISPR/Cas12a system. Multiple sequence alignment of the *TUB2* gene fragment revealed that *C. pyri* and the congeneric *Cytospora* species exhibited high sequence conservation within this region, while harboring a sufficient number of discriminatory nucleotide polymorphisms (Figure 1). The LAMP primer-binding regions—F3, F2, F1c, B1c, B2, B3, and LF—were unambiguously mapped onto the aligned sequences, and the continuity of these regions in the target strain sequences was adequate to support subsequent LAMP, which was empirically validated in Section 3.2.

Multiple sequence alignment of partial *TUB2* gene sequences from *C. pyri* and related congeneric *Cytospora* species. Black arrows indicate LAMP primer-binding regions (F3, F2, F1c, B1c, B2, B3, and loop primer LF). Red boxes and red arrows denote candidate Cas12a crRNA target sites. Sequence shading indicates the degree of conservation, with dark shading representing highly conserved regions and light/unshaded positions indicating nucleotide variations among species. Schematic illustration of the LAMP primer arrangement and crRNA target sites within the selected *TUB2* fragment (Figure 1).

A single LAMP primer set was selected, comprising two outer primers (F3 and B3), two inner primers (FIP and BIP), and one loop primer (LF) (Table 2). The FIP primer was chimeric, consisting of F1c and F2 sequences; similarly, BIP comprised B1c and B2 sequences. Due to the limited availability of suitable regions for loop primer arrangement within the *TUB2* target fragment, only one loop primer (LF) was retained. Concurrently, two candidate Cas12a crRNA recognition sites were identified within the LAMP-amplified region (both located in the vicinity of the F2/B1c region) and were selected for CRISPR/Cas12a fluorescence detection (Table 3). These results demonstrate that the *TUB2* target region is amenable to simultaneous LAMP primer design and Cas12a crRNA target site selection, providing the molecular foundation for construction of the hybrid LAMP-CRISPR/Cas12a detection system.

### 3.2. Optimization of LAMP Reaction Conditions

To establish a robust and efficient LAMP system, reaction temperature, reaction time, primer concentration ratio, and final dNTP concentration were systematically optimized.

**Figure 2:**
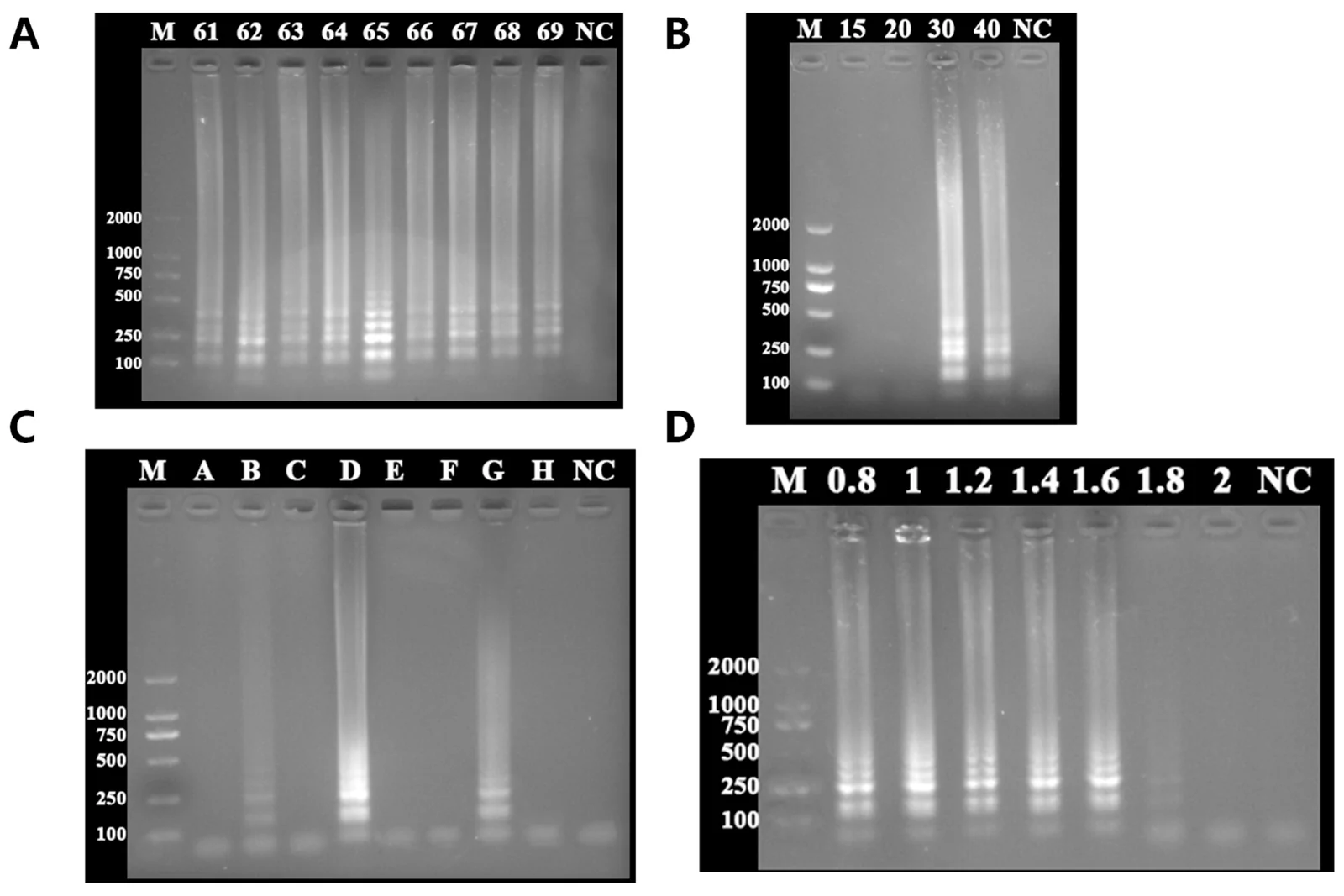
Optimization of LAMP reaction conditions. (**A**) Temperature optimization. LAMP reactions were performed at 61–69 °C for 30 min. M, DNA 2K Plus Marker; lanes 61–69, reaction temperatures of 61, 62, 63, 64, 65, 66, 67, 68, and 69 °C, respectively; NC, negative control (Nuclease-free water). (**B**) Time optimization at 65 °C. M, DNA 2K Plus Marker; lanes 15, 20, 30, and 40, reaction times of 15, 20, 30, and 40 min, respectively; NC, negative control. (**C**) Primer concentration ratio optimization at 65 °C for 30 min. M, DNA 2K Plus Marker; lanes A–H, outer:inner:loop primer ratios of 1:6:2, 1:6:3, 1:6:4, 1:8:2, 1:8:3, 1:8:4, 2:8:3, and 2:6:3, respectively; NC, negative control. (**D**) dNTP final concentration optimization at 65 °C for 30 min. M, DNA 2K Plus Marker; lanes 0.8–2, dNTP concentrations of 0.8, 1.0, 1.2, 1.4, 1.6, 1.8, and 2.0 mM, respectively; NC, negative control.

Amplification signals were detectable across the entire temperature range 61–69 °C, albeit with varying intensities. At 65 °C, the amplification products exhibited clear, continuous ladder-like bands with substantially higher band intensity than at other temperatures, while no amplification was visible in the negative control (Figure 2A). Accordingly, 65 °C was selected as the optimal reaction temperature for subsequent LAMP assays.

The effect of reaction duration at 65 °C was then evaluated. No discernible amplification bands were observed after 15 and 20 min, but distinct bands emerged after 30 min, with intensity comparable to the 40 min reactions (Figure 2B). Balancing amplification efficiency and detection turnaround time, 30 min was designated the optimal reaction duration.

The relative proportions of outer, inner, and loop primers substantially influenced amplification product formation. Of the eight tested ratios, 1:8:2 produced the most distinct bands with the strongest amplification signal, while no amplification was evident in the negative control (Figure 2C). Therefore, an outer:inner:loop primer ratio of 1:8:2 was established as optimal.

Amplification signals were generated across the final dNTP concentration range of 0.8 to 1.6 mM. dNTP at 1.4 mM produced clear bands with minimal background while amplification was markedly attenuated at 1.8 and 2.0 mM (Figure 2D), so 1.4 mM was selected as the optimal final dNTP concentration.

Thus, as detailed in Table 4, the optimum LAMP reaction conditions were: reaction temperature 65 °C, reaction time 30 min, outer:inner:loop primer ratio 1:8:2, and final dNTP concentration 1.4 mM.

### 3.3. Optimization of CRISPR/Cas12a Reaction Conditions

To establish stable and reproducible CRISPR/Cas12a fluorescence detection conditions, two candidate crRNAs (crRNA-1 and crRNA-2) were evaluated, followed by systematic optimization of reaction temperature, reaction time, LbCas12a concentration, LbCas12a:crRNA molar ratio, and ssDNA reporter concentration.

**Figure 3:**
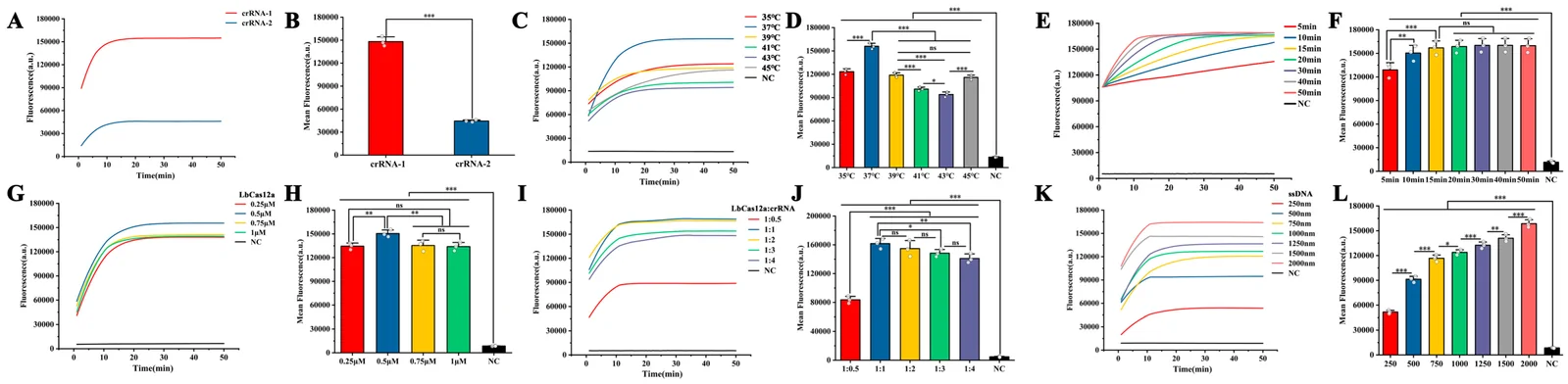
Optimization of CRISPR/Cas12a reaction conditions. (**A**,**B**) crRNA screening. (**A**) Real-time fluorescence curves of crRNA-1 and crRNA-2. (**B**) Endpoint fluorescence intensity. (**C**,**D**) Temperature optimization. (**C**) Real-time fluorescence curves at 35–45 °C. (**D**) Endpoint fluorescence intensity. (**E**,**F**) Time optimization at 37 °C. (**E**) Real-time fluorescence curves at 5–50 min. (**F**) Endpoint fluorescence intensity. (**G**,**H**) LbCas12a concentration optimization at 37 °C for 15 min. (**G**) Real-time fluorescence curves at 0.25–1.00 μM. (**H**) Endpoint fluorescence intensity. (**I**,**J**) LbCas12a:crRNA molar ratio optimization at 37 °C for 15 min. (**I**) Real-time fluorescence curves at ratios of 1:0.5–1:4. (**J**) Endpoint fluorescence intensity. (**K**,**L**) ssDNA reporter concentration optimization at 37 °C for 15 min. (**K**) Real-time fluorescence curves at 250–2000 nM. (**L**) Endpoint fluorescence intensity. Data are presented as mean ± SD from three independent replicates. NC, negative control (nuclease-free water); * *p* < 0.05, ** *p* < 0.01, *** *p* < 0.001; ns, not significant.

crRNA-1 produced a more rapid fluorescence curve elevation and higher endpoint fluorescence intensity than crRNA-2, with a signal response markedly superior to the negative control (Figure 3A,B). Accordingly, crRNA-1 was selected and used as the standard recognition element in all subsequent experiments (specificity, sensitivity, strain applicability, and field-sample analysis).

Fluorescence signals were detectable under all the tested temperatures; however, 37 °C produced the most rapid fluorescence curve elevation and the highest endpoint intensity (Figure 3C,D). Thus, 37 °C was selected as the optimal reaction temperature.

Reaction time analysis at 37 °C showed that fluorescence was detectable after 5 min, albeit with relatively low endpoint intensity. Fluorescence strength increased substantially by 10 min and plateaued after 15 min, with minimal variation in endpoint intensity from 15 to 50 min (Figure 3E,F). Balancing detection efficiency and reaction duration, 15 min was designated the optimal reaction time.

LbCas12a at 0.5 μM produced the strongest fluorescence response (endpoint intensity exceeded the 0.25, 0.75, and 1.0 μM conditions) and the negative control maintained consistently low background (Figure 3G,H), so 0.5 μM was established as the optimum LbCas12a concentration.

The 1:1 LbCas12a:crRNA molar ratio produced the highest fluorescence signal. While the 1:2, 1:3, and 1:4 ratios also generated fluorescence responses, their endpoint intensities were inferior. Since the 1:0.5 ratio also produced a weaker signal (Figure 3I,J), an LbCas12a:crRNA molar ratio of 1:1 was selected as the optimum.

ssDNA reporter concentration produced a concentration-dependent increase in fluorescence, with 2000 nM producing the highest endpoint intensity and negligible background in the negative control (Figure 3K,L). Consequently, 2000 nM was selected as the optimal ssDNA reporter concentration.

Thus, as detailed in Table 5, the optimum CRISPR/Cas12a reaction conditions were: crRNA-1 recognition element, reaction temperature 37 °C, reaction time 15 min, LbCas12a concentration 0.5 μM, LbCas12a:crRNA molar ratio 1:1, and ssDNA reporter concentration 2000 nM.

### 3.4. Validation of the Specificity of the LAMP-CRISPR/Cas12a System

To evaluate the detection specificity of the system for *Cytospora* pathogens associated with pear canker, a panel of fungal isolates comprising *C. pyri*, the closely related species *C. mali*, other *Cytospora* species, and non-target fungi was subjected to the optimized LAMP-CRISPR/Cas12a assay.

**Figure 4:**
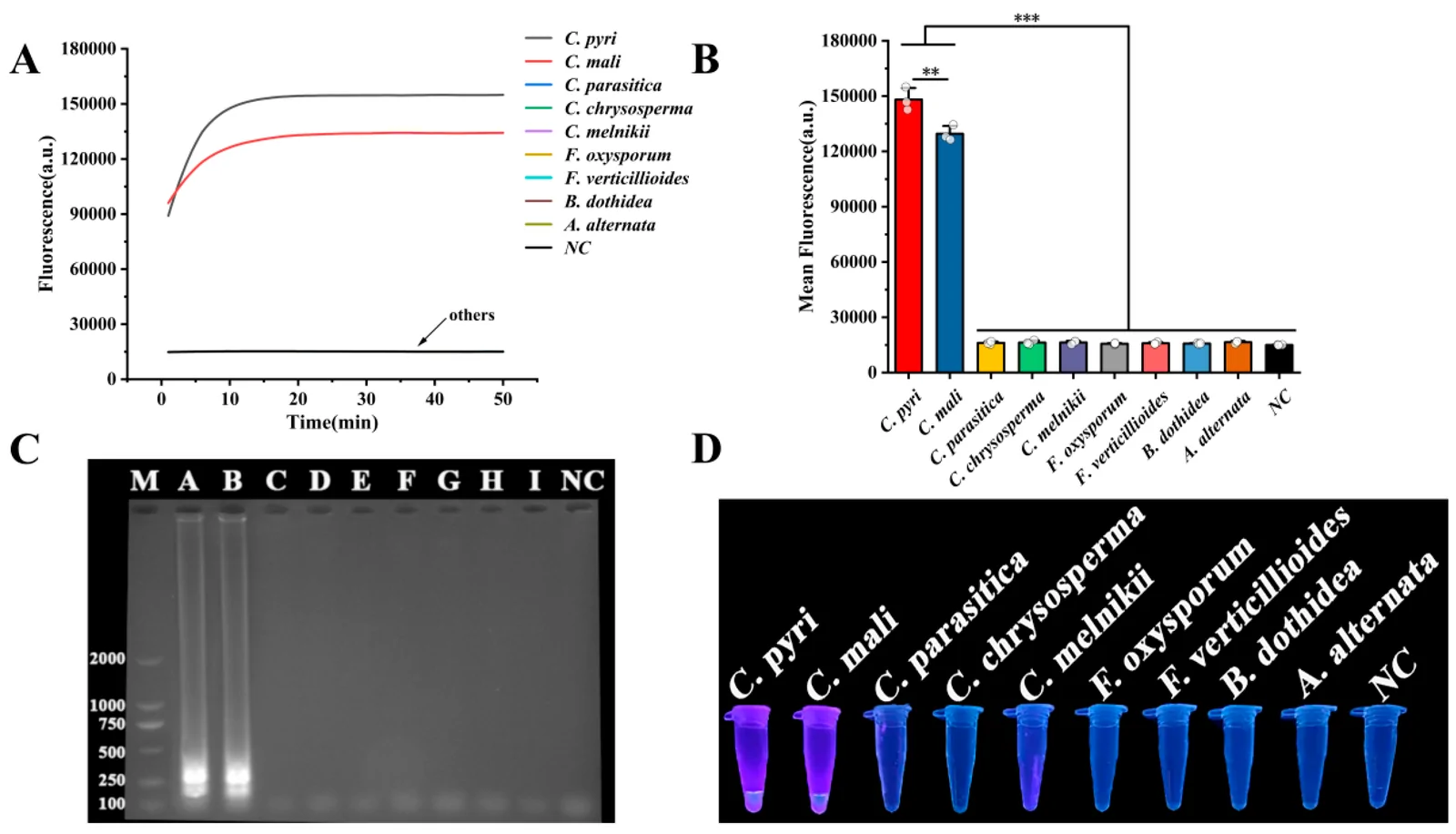
Specificity evaluation of the LAMP-CRISPR/Cas12a system. (**A**) Real-time fluorescence curves of CRISPR/Cas12a detection. (**B**) Endpoint fluorescence intensity. Data are mean ± SD from three independent replicates; ** *p* < 0.01, *** *p* < 0.001. (**C**) LAMP products analyzed by 1.2% agarose gel electrophoresis. (**D**) UV-assisted visual detection of CRISPR/Cas12a fluorescence following LAMP amplification. Strains tested: 1, *C. pyri*; 2, *C. mali*; 3, *C. parasitica*; 4, *C. chrysosperma*; 5, *C. melnikii*; 6, *F. oxysporum*; 7, *F. verticillioides*; 8, *Botryosphaeria dothidea*; 9, *A. alternata*; NC, negative control (nuclease-free water).

Both *C. pyri* and *C. mali* generated robust signals under CRISPR/Cas12a real-time fluorescence analysis, with curves exhibiting rapid ascent and plateauing within the reaction window. By contrast, all other tested strains and the negative control produced only consistently low background fluorescence (Figure 4A). This was consistent with the observed endpoint fluorescence intensities, with *C. pyri* and *C. mali* exhibiting significantly higher fluorescence than the other strains and the negative control (Figure 4B).

LAMP product analysis by gel electrophoresis demonstrated that characteristic bands were observed exclusively for *C. pyri* and *C. mali*, while no products were detected with the other strains or negative control (Figure 4C). Subsequent CRISPR/Cas12a-mediated visual examination under UV light confirmed that *C. pyri* and *C. mali* reaction tubes displayed intense green fluorescence, whereas the other strains and the negative control did not fluoresce (Figure 4D).

These results show that the LAMP-CRISPR/Cas12a system specifically detects *C. pyri* and its closely related congener *C. mali*, discriminating these target pathogens from other tested *Cytospora* species and non-target fungi. However, it cannot differentiate between *C. pyri* and *C. mali*; rather, it is most suited for rapid screening for the *Cytospora* pathogens associated with pear canker.

### 3.5. Validation of the Sensitivity of the LAMP-CRISPR/Cas12a System

To determine the analytical sensitivity of the system, serial dilutions of *C. pyri* genomic DNA ranging from 10^5^ to 10^−3^ copies/μL were subjected to the optimized LAMP-CRISPR/Cas12a assay.

**Figure 5:**
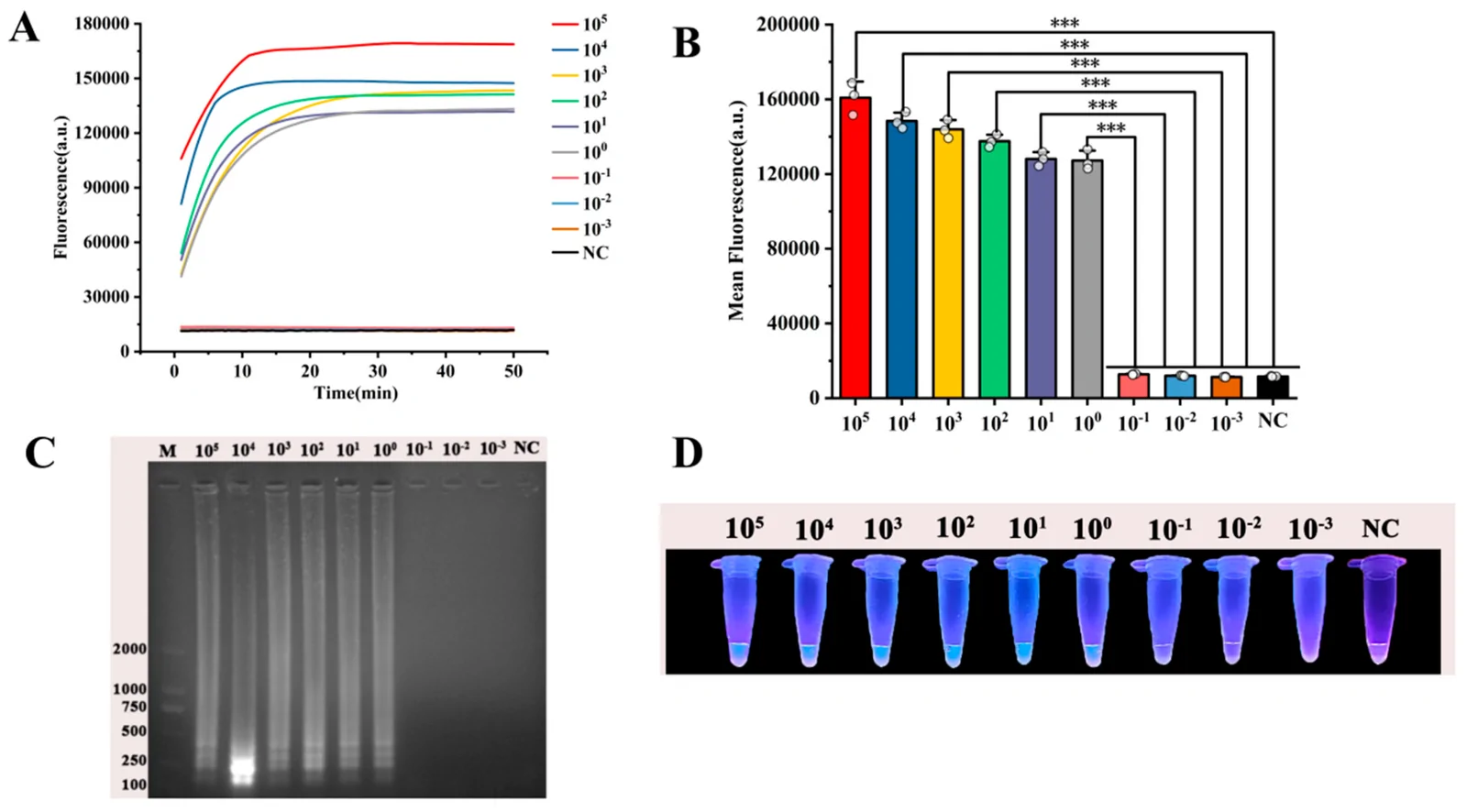
Sensitivity analysis of the LAMP-CRISPR/Cas12a system. (**A**) CRISPR/Cas12a real-time fluorescence curves for serial dilutions of *C. pyri* genomic DNA. (**B**) Endpoint fluorescence intensity. Data are mean ± SD from three independent replicates. (**C**) LAMP amplification products analyzed using 1.2% agarose gel electrophoresis. (**D**) UV-assisted visual detection of CRISPR/Cas12a fluorescence following LAMP amplification using template concentrations from 10^5^ to 10^−3^ copies/μL; NC, negative control (nuclease-free water). Data in (**B**) are presented as mean ± SD from three independent replicates. *** *p* < 0.001.

Real-time fluorescence detection produced robust signals across the template concentration range 10^5^–10^0^ copies/μL, with curves exhibiting rapid ascent and progressive plateauing. By contrast, fluorescence signals at 10^−1^ copies/μL and below approximated the background of the negative control (Figure 5A). This was consistent with the endpoint fluorescence intensities, with the 10^5^–10^0^ copies/μL groups exhibiting significantly higher fluorescence than the negative control, while the 10^−1^, 10^−2^, and 10^−3^ copies/μL groups did not generate discernible signals (Figure 5B).

LAMP product analysis by gel electrophoresis showed characteristic bands with the 10^5^–10^0^ copies/μL template concentrations. No amplification products were observed at 10^−1^ copies/μL or below, or in the negative control (Figure 5C). Subsequent CRISPR/Cas12a-mediated UV-assisted visual examination confirmed that fluorescence was visible in the 10^5^–10^0^ copies/μL groups, but not at 10^−1^ copies/μL or below (Figure 5D).

These results demonstrate that, within the tested concentration range, the LAMP-CRISPR/Cas12a system consistently detects target templates as low as 10^0^ copies/μL, establishing the LOD at 1 copy/μL.

### 3.6. Applicability of the LAMP-CRISPR/Cas12a System Across Diverse Cytospora Strains

To evaluate the applicability of the system across diverse *Cytospora* isolates, eight *Cytospora* isolates previously identified as pathogens associated with pear canker were subjected to the optimized assay.

**Figure 6:**
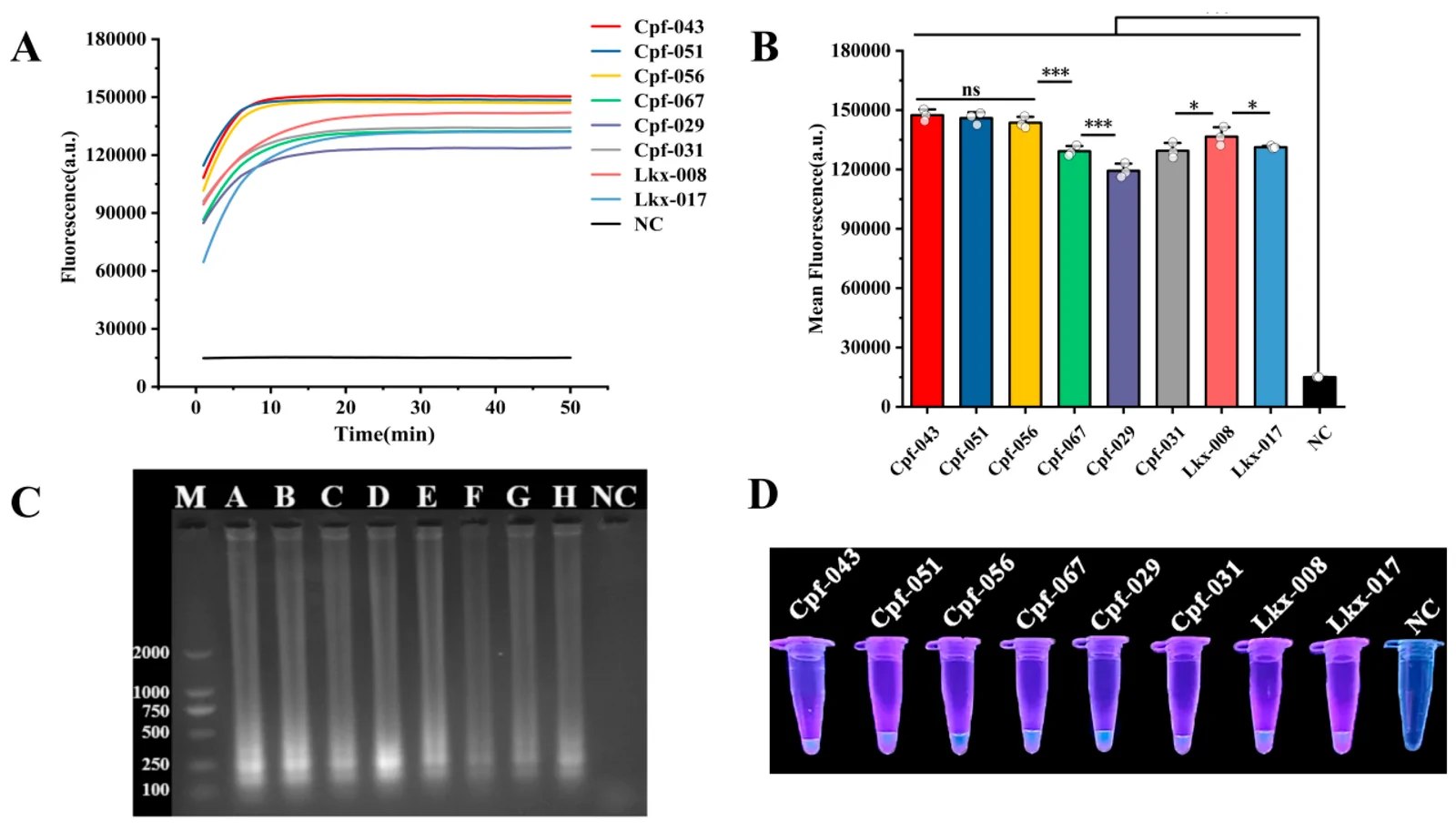
Applicability of the LAMP-CRISPR/Cas12a system to strains from diverse origins. (**A**) Real-time fluorescence curves of CRISPR/Cas12a detection. (**B**) Endpoint fluorescence intensity. (**C**) LAMP products analyzed by 1.2% agarose gel electrophoresis. (**D**) UV-assisted visual detection of CRISPR/Cas12a fluorescence following LAMP amplification. M, DNA 2K Plus Marker; lanes A–H, *Cytospora* strains Cpf-043, Cpf-051, Cpf-056, Cpf-067, Cpf-029, Cpf-031, Lkx-008, and Lkx-017, respectively; NC, negative control (Nuclease-free water). Data in (**B**) are presented as mean ± SD from three independent replicates. ns, not significant; * *p* < 0.05, and *** *p* < 0.001.

All eight strains generated robust CRISPR/Cas12a real-time fluorescence signals, with curves exhibiting rapid ascent and progressive plateauing, while the negative control produced consistently low fluorescence throughout the reaction (Figure 6A). All eight strains also exhibited significantly higher endpoint fluorescence intensity than the negative control (Figure 6B).

LAMP product analysis by gel electrophoresis showed characteristic bands for all eight strains, while no amplification products were discernible in the negative control (Figure 6C). Subsequent CRISPR/Cas12a-mediated UV-assisted visual examination confirmed fluorescence was visible in all eight strains but not in the negative control (Figure 6D).

These findings illustrate the agreement between LAMP gel electrophoresis, CRISPR/Cas12a real-time fluorescence detection, and UV-assisted visual assessment, demonstrating that this system for the detection of *Cytospora* pathogens associated with pear canker can be applied to strains from diverse geographic and host origins.

### 3.7. Analysis of Field Samples

**Figure 7:**
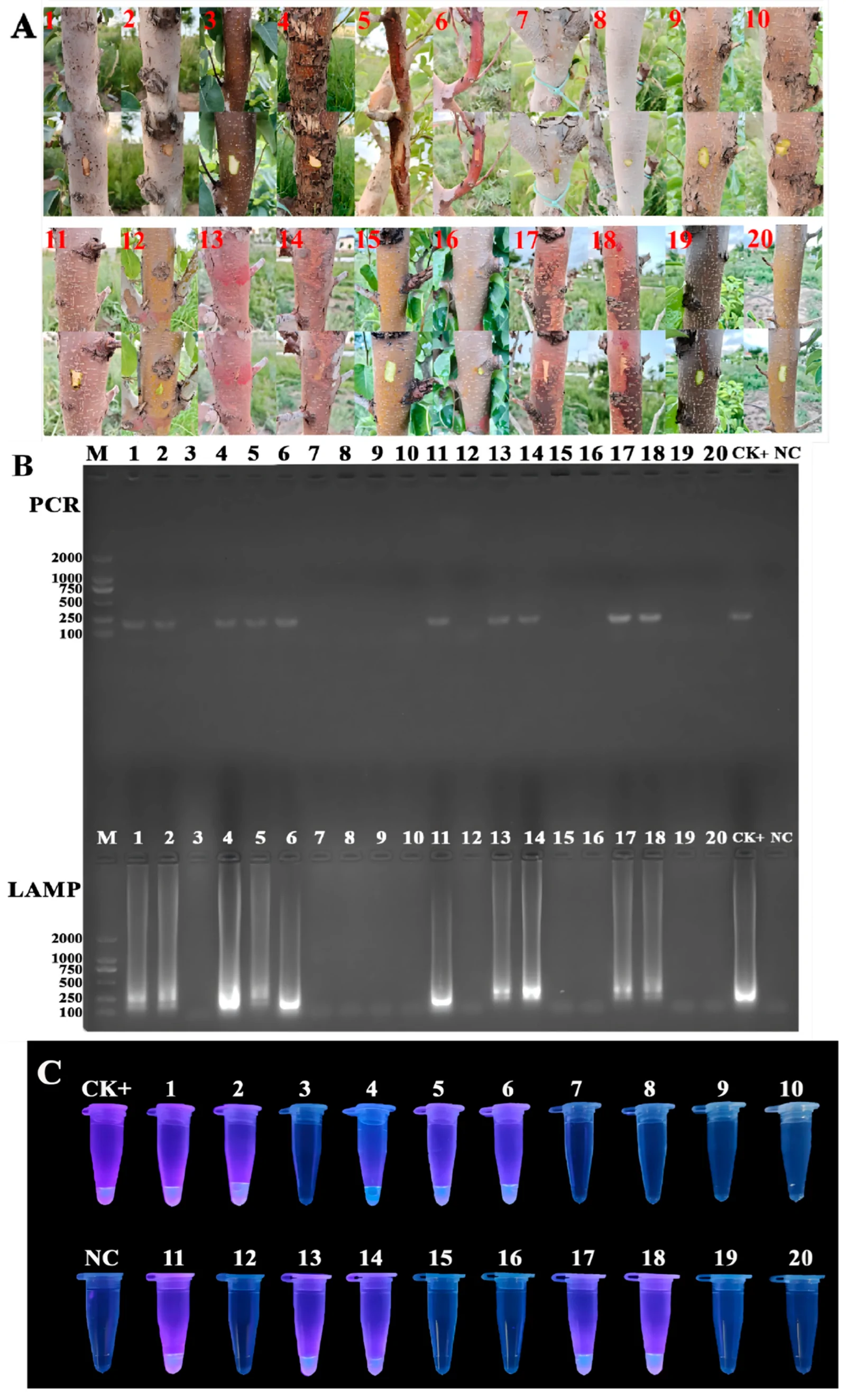
Detection of target pathogens in field-collected pear canker samples. (**A**) Field appearance of 20 fragrant pear branch samples collected from commercial orchards. Samples 1, 2, 4, 5, 14, 17, and 18 were classified as symptomatic; samples 11 and 13 as suspected; and samples 3, 6, 7, 8, 9, 10, 12, 15, 16, 19, and 20 as asymptomatic. (**B**) Conventional PCR and LAMP results following DNA extraction using the modified PEG–sodium acetate precipitation method. (**C**) UV-assisted visual detection of CRISPR/Cas12a fluorescence following LAMP amplification. M, DNA 2K Plus Marker; CK+, positive control (*C. pyri* DNA); NC, negative control (nuclease-free water).

To assess the applicability of the LAMP-CRISPR/Cas12a system to real-world orchard samples, 20 branch tissue samples (one per tree; symptom status illustrated in Figure 7A) from fragrant pear trees in commercial orchards were subjected to parallel analysis by conventional PCR, LAMP, and LAMP-CRISPR/Cas12a assays; the symptomatic status of each sample is indicated in Figure 7A. The positive control (CK+) produced positive results under all three assays, while the negative control produced no amplification or fluorescence signals, confirming the operational integrity of the detection systems.

Conventional PCR analysis (Figure 7B) produced target bands in samples 1, 2, 4, 5, 6, 11, 13, 14, 17, and 18, and no discernible amplification in the remaining samples. LAMP analysis results were consistent with PCR: the aforementioned PCR-positive samples exhibited characteristic bands, while PCR-negative samples showed no amplification under LAMP. Subsequent CRISPR/Cas12a visual fluorescence evaluation confirmed intense fluorescence signals in the PCR-positive samples and no fluorescence in the PCR-negative samples and the negative control.

These results demonstrate that the LAMP-CRISPR/Cas12a system can be applied to the rapid screening of target pathogens in real-world pear branch samples, with detection outcomes consistent with conventional PCR.

## 4. Discussion

### 4.1. Description and Performance of the LAMP-CRISPR/Cas12a System

To address the limitations of traditional approaches to diagnosing fruit tree canker diseases—namely, protracted detection cycles, limited sensitivity to low-abundance pathogens, and potential non-specific amplification in standalone LAMP assays—this study developed a hybrid LAMP-CRISPR/Cas12a detection system that incorporates a simple visual assessment and targets *Cytospora* pathogens associated with pear canker. *C. pyri* and its closely related congener *C. mali* (the latent pathogens responsible for pear canker) are the primary target pathogens and the system utilizes LAMP primers and Cas12a crRNA aimed at the *TUB2* target region. It enriches target nucleic acids via 30-min LAMP at 65 °C, followed by a 15-min CRISPR/Cas12a reaction at 37 °C that recognizes amplification products and generates fluorescence signals. When applied to DNA extracted from a sample, the amplification and detection stages can be completed within approximately 45 min. This hybrid system can reliably detect as few as 10^0^ copies/μL of target template. Results can be interpreted via real-time fluorescence curves, endpoint fluorescence intensity, and UV-assisted visual examination, demonstrating its potential for application in both laboratory testing and rapid screening of samples in the field. The primary practical benefit of this assay is its combination of high analytical sensitivity with sequence-specific confirmation of the LAMP amplicon, which reduces the risk of false-positive visual readings that could compromise diagnosis in field settings. This is particularly relevant with latently infected tissues in which low pathogen biomass demands both high sensitivity and high specificity to avoid misdiagnosis.

### 4.2. Comparison with Previously Reported Methods

Compared with previously reported LAMP-only methods, this LAMP-CRISPR/Cas12a system offers a relatively short reaction time and an additional sequence-recognition step. Xu et al. [11] established a LAMP detection method for *C. mali* with a reaction time of 60 min and a LOD of approximately 10 copies/μL. The present study optimized a LAMP primer set and reaction system so the amplification phase could be reduced to 30 min, while also introducing Cas12a-crRNA recognition and ssDNA reporter fluorescence modules. Although the CRISPR/Cas12a detection step adds approximately 15 min to the analysis time, the amplification and detection phases can be completed within 45 min. This hybrid approach, with its short reaction time and sequence recognition of amplification products, gives greater certainty to result interpretation. However, it should be noted that the 45-min timing refers to the assay reaction time only and does not include DNA extraction, which can represent a substantial portion of the overall diagnostic turnaround time, particularly for woody tissues under field conditions. In addition, because this study did not perform a systematic head-to-head comparison with established methods (e.g., qPCR and conventional PCR), the data demonstrate feasibility and concordance with conventional PCR rather than a general claim of method superiority.

### 4.3. Rationale for CRISPR/Cas12a as a Secondary Specificity Safeguard

The main purpose of introducing the CRISPR/Cas12a module was to provide a second, sequence-specific recognition step that confirms the identity of the LAMP amplicon, thereby reducing the risk of misinterpreting non-specific amplification products generated in standalone LAMP assays. The LAMP reaction uses multiple primer pairs to recognize various regions of the target sequence. This allows high amplification efficiencies and reaction speeds, but the multiple primers can also increase the likelihood of primer–dimer formation and non-specific binding. Such non-specific amplification can affect interpretation of results, particularly in high-sensitivity assays [45]. The present study incorporates a Cas12a-crRNA recognition step after the LAMP. When the amplification product contains a target sequence that matches the crRNA, it activates the trans-cleavage activity of Cas12a, which cleaves the ssDNA reporter and triggers a fluorescence signal. Specificity analysis showed this system produced positive fluorescence signals in the presence of *C. pyri* or its closely related species *C. mali,* but not in the presence of various other *Cytospora* species or other common fungi pathogenic to fruit trees. This indicates that the system can be used for rapid detection of *Cytospora* pathogens associated with pear canker. It should be acknowledged, however, that the specificity experiment evaluated the combined LAMP-CRISPR/Cas12a system as a whole; it did not, by itself, quantitatively demonstrate that Cas12a eliminated false-positive products generated by LAMP alone. Comparative data (LAMP vs. LAMP-CRISPR/Cas12a) would be required to establish the contribution of the Cas12a module to the combined system.

### 4.4. Strategic Design for Detection of Congeneric Pathogens

The intended diagnostic target of this assay was the group of dominant *Cytospora* pathogens associated with pear canker—in particular *C. pyri* and its closely related congener *C. mali*—rather than a single species. The LAMP-CRISPR/Cas12a system is not designed to distinguish between *C. pyri* and *C. mali* at the species level. LAMP and CRISPR/Cas12a detection produced positive responses for both pathogens. This is presumably due to the selected *TUB2* target region being relatively conserved between these two closely related species and, consequently, the crRNA sites being insufficient to achieve species-level resolution. This target selection was a deliberate choice, based on the pathogens’ cross-host infection characteristics, the core requirements for disease control and prevention in the field, and the cost constraints of large-scale field screening, given that both *C. pyri* and *C. mali* are closely associated with branch and trunk canker diseases of fruit trees.

From a technical perspective, differentiation of *C. pyri* and *C. mali* could be achieved by screening for stable, species-specific differential sites and designing species-specific crRNA or multiplex crRNA typing systems. However, in the context of large-scale screening of orchard trees, focusing on single-species detection could risk missing closely related pathogens. Both *C. pyri* and *C. mali* are closely associated with branch and trunk canker diseases in fruit trees, and closely related *Cytospora*/*Valsa* pathogens may co-exist in samples from different hosts and regions [46,47,48]. Therefore, given the practical requirements for controlling pear canker, it is reasonable to adopt a screening strategy targeting conserved regions capable of simultaneously detecting both *C. pyri* and *C. mali*. This not only reduces the risk of missed detections associated with single-species targets but also avoids increasing the complexity of the test and the number of operational steps. This ensures the system is suitable for rapid testing of orchard samples and preliminary screening of seedlings for the presence of pathogens. More complex applications, such as pathogen population monitoring and epidemiological surveys, will require integration of dual-crRNA genotyping systems, species-specific PCR, or sequencing analysis to achieve species-level identification.

### 4.5. Current Limitations and Future Directions

The performance data we report define both the scope and the boundaries of the LAMP-CRISPR/Cas12a system. Specificity experiments showed the assay detects *C. pyri* and its closely related congener *C. mali*, while producing no signal for the other *Cytospora* species and common fruit-tree pathogenic fungi that were tested; it should, therefore, be regarded as a group-level screening tool for the dominant pear canker pathogens rather than a general fungal diagnostic tool. Its usefulness for other plant fungal pathogens has not been established and should not be inferred from the present data, although the limited size and diversity of the specificity panel leave room for broader cross-reactivity testing. Two design-related constraints follow directly from the results. First, Cas12a recognition requires a TTTV-type PAM, which restricted the target-site selection within the TUB2 gene. Second, because this region is conserved between *C. pyri* and *C. mali*, the system cannot discriminate between these species. This is acceptable for screening purposes but precludes its use in species-level epidemiological studies. The two-stage protocol also requires the reaction tube to be opened to add the CRISPR reagents after LAMP amplification, posing a risk of aerosol contamination. Sealed single-tube reaction formats should be evaluated to eliminate this risk before adoption for routine use [40]. Several additional limitations are also evident: (i) the field evaluation was restricted to 20 samples without quantitative comparison with qPCR or another established reference method, (ii) potential effects of inhibitors in pear bark and woody tissues, although mitigated by the modified PEG–sodium acetate extraction, were not directly quantified, (iii) independent multi-locus sequence data for all target isolates have not yet been publicly deposited, and (iv) inter-laboratory validation has not been performed. Future studies should enlarge the isolate and field sample sets, employ quantitative benchmarking against qPCR, evaluate sealed-tube reaction formats and dual-mode visualization methods that combine colorimetric LAMP indicators (e.g., hydroxynaphthol blue [49,50] and pH-sensitive dyes [51,52]) with CRISPR fluorescence confirmation, and extend testing to more woody-plant hosts and other canker-associated pathogens to determine the extent of the applicability of this assay.

## 5. Conclusions

This study demonstrates that a hybrid LAMP-CRISPR/Cas12a format is a feasible route to rapid, sequence-verified screening of the dominant *Cytospora* canker pathogens found in Korla fragrant pear orchards. By coupling isothermal amplification with programmable, sequence-specific confirmation in an instrument-light workflow, this approach addresses the diagnostic gap in symptom-based management, which cannot detect the latent infections that drive disease spread. Within defined scope and boundaries, this system offers a practical basis for early warning, pathogen surveillance, and precision management of pear canker.

## Figures and Tables

**Table 1:** Fungal strains analyzed in this study.

Strains	Species	Host	Origin	LAMP ^a^	CRISPR/Cas12a ^a^	GenBank Accession Number
Cpf-043	*Cytospora pyri*	Pear tree	Kashi, Xinjiang Uygur, China	+	+	PZ629081
Cpf-051	*C. pyri*	Pear tree	Kashi, Xinjiang Uygur, China	+	+	PZ629082
Cpf-056	*C. pyri*	Pear tree	Kashi, Xinjiang Uygur, China	+	+	PZ629083
Cpf-067	*C. pyri*	Pear tree	Kashi, Xinjiang Uygur, China	+	+	PZ629084
Cpf-069	*C. pyri*	Pear tree	Kashi, Xinjiang Uygur, China	+	+	PZ629085
Cpf-074	*C. pyri*	Pear tree	Kashi, Xinjiang Uygur, China	+	+	PZ629086
Cpf-087	*C. pyri*	Pear tree	Kashi, Xinjiang Uygur, China	+	+	PZ629087
Cpf-094	*C. pyri*	Pear tree	Kashi, Xinjiang Uygur, China	+	+	PZ629088
Cpf-104	*C. pyri*	Pear tree	Kashi, Xinjiang Uygur, China	+	+	PZ629089
Cpf-106	*C. pyri*	Pear tree	Kashi, Xinjiang Uygur, China	+	+	PZ629090
Cpf-127	*C. pyri*	Pear tree	Kashi, Xinjiang Uygur, China	+	+	PZ629091
Cpf-139	*C. pyri*	Pear tree	Kashi, Xinjiang Uygur, China	+	+	PZ629092
Cpf-148	*C. pyri*	Pear tree	Aral, Xinjiang Uygur, China	+	+	PZ629093
Cpf-149	*C. pyri*	Pear tree	Aral, Xinjiang Uygur, China	+	+	PZ629094
Cpf-161	*C. pyri*	Pear tree	Aral, Xinjiang Uygur, China	+	+	PZ629095
Cpf-168	*C. pyri*	Pear tree	Aral, Xinjiang Uygur, China	+	+	PZ629096
Cpf-187	*C. pyri*	Pear tree	Aral, Xinjiang Uygur, China	+	+	PZ629097
Cpf-193	*C. pyri*	Pear tree	Aral, Xinjiang Uygur, China	+	+	PZ629098
Cpf-207	*C. pyri*	Pear tree	Aral, Xinjiang Uygur, China	+	+	PZ629099
Cpf-214	*C. pyri*	Pear tree	Aral, Xinjiang Uygur, China	+	+	PZ629100
Cpf-029	*Cytospora mali*	Pear tree	Kashi, Xinjiang Uygur, China	+	+	PZ629101
Cpf-031	*C. mali*	Pear tree	Kashi, Xinjiang Uygur, China	+	+	PZ629102
Cpf-038	*C. parasitica*	Pear tree	Kashi, Xinjiang Uygur, China	-	-	PZ674754
Lkx-008	*C. mali*	Apple tree	Aral, Xinjiang Uygur, China	+	+	PZ629103
Lkx-017	*C. mali*	Apple tree	Aral, Xinjiang Uygur, China	+	+	PZ629104
Lkx-031	*C. mali*	Apple tree	Aral, Xinjiang Uygur, China	+	+	PZ629105
Lkx-006	*C. chrysosperma*	Apple tree	Kashi, Xinjiang Uygur, China	-	-	PZ674755
Lkx-013	*C. melnikii*	Apple tree	Kashi, Xinjiang Uygur, China	-	-	PZ674756
Lkx-027	*Fusarium oxysporum*	Apple tree	Kashi, Xinjiang Uygur, China	-	-	PZ674757
Lkx-029	*Fusarium verticillioides*	Apple tree	Kashi, Xinjiang Uygur, China	-	-	PZ674758
BotAJ-065	*Botryosphaeria dothidea*	Albizia julibrissin	Kashi, Xinjiang Uygur, China	-	-	PX238467
AltPD-091	*A. alternata*	Prunus domestica	Kashi, Xinjiang Uygur, China	-	-	PX229955

a “+” indicates a positive reaction, whereas “-” indicates a negative reaction.

**Table 2:** Sequences of LAMP primers.

Name	Sequence (5′-3′)
LAMP-F3	ATGGCCATCATCTCGAAACA
LAMP-B3	TCGAGCTGGAGCTCAGAA
LAMP-FIP	CGGGGGTTGGGAGGTACATACAACAGGCAAACCATCTCTGG
LAMP-BIP	ACGCCCTTTCGCGTTGCTTCGTGCCGTTGTAACTGTGTGA
LAMP-LF	TCCATTGCTGTCGAGACCGT

**Table 3:** Cas12a crRNA and ssDNA reporter sequences.

Name	Sequence (5′-3′)
crRNA-1	AAUUUCUACUAAGUGUAGAUGCGUUGCUUCUAGCCUUGUAUU
crRNA-2	AAUUUCUACUAAGUGUAGAUCCUGUAGUCGAGGUCAGUAUUC
ssDNA	5′FAM-TTTTTTTTTTTT-3′BHQ

**Table 4:** Optimized LAMP reaction composition.

Serial Number	Reaction Reagent	Added Volume (μL)
1	10 × Isothermal LAMP Buffer	2.5
2	dNTPs (10 mM)	3.5
3	10 × LAMP Primer Mix	2.5
4	Bst 2.0 DNA polymerase (8 U/μL)	1.0
5	Betaine (5M)	2.0
6	DNA	3.0
7	MgSO_4_ (100 mM)	1.5
8	Nuclease-free water	9.0
9	Total	25.0

**Table 5:** Optimized CRISPR/Cas12a reaction composition.

Serial Number	Reaction Reagent	Volume (μL)
1	10 × Cleavage Buffer	2.0
2	LbCas12a Nuclease (10 μM)	1.0
3	crRNA (10 μM)	1.0
4	ssDNA (10 μM)	4.0
5	LAMP products	2.0
6	Nuclease-free water	10.0
7	Total	20.0

## Data Availability

The original contributions presented in this study are included in the article. Further inquiries can be directed to the corresponding authors.
